# Dr. Kinglake on Morbid Sensation

**Published:** 1812-12

**Authors:** Robert Kinglake

**Affiliations:** Taunton


*To the Editors of the Medical and Physical Journal.*

GENTLEMEN,

THAT a function so important as that which imparts life and consciousness to animal nature, should be liable to various diseases, is too obvious to be insisted on. It is, indeed, rather a subject of astonishment than otherwise, that a frame so nicely constituted as the animal fabric is, should be capable of existing with so few disorders as really occur. The more evident forms of disease are denoted in characters too striking to be mistaken; hence they are too familiar to common observation ever to have their existence and aspect disputed; but in the diseases of sensation the symptoms are transient, and leave but faint traces of their existence and form. Sensation is to the nervous, what irritation is to the muscular, structure; these are respectively the functions of peculiar peculiar organisation, in which are inherent, or are evolved, the vital conditions of sensibility and irritability, the faculties or sources of the sentient and irritative actions of life.

The vital condition denominated sensibility is a faculty as it were superadded to that of irritability, which universally pervades the animal frame. Not a living motion could occur without the irritative power, but the sensitive action is not equally indispensible to animal existence. The grand purpose of sensibility is to afford to animal being consciousness, and thus to contra-distinguish it from the subjects of vegetative organisation. The brain and all its distributions, collectively called the nervous system, are therefore necessary to a knowledge of existence and of surrounding nature; and without them nothing could be imagined, felt, or understood. To the existence then of this power, and to the infinite combinations of its influence, all consciousness and intelligence are to be ascribed.

Both the sensitive and irritative functions of animal life have their respective spheres of action, which cannot be transgressed without creating disorder. An absolute or relative excess or deficiency of either, will be discovered in corresponding symptoms of disease. In an excess of sensibility, much irregular and even afflicting sensation will arise; whilst, in that of irritability, hurtful and even destructive conflicts may be going on. In morbid sensation, it is necessary clearly to shew how it arises, and in what it precisely consists, when it will be easy to trace its variations, and to refer its anomalies to a diseased state of that vital function. Whatever may have had the effect of disordering the healthful connection naturally subsisting between the several functions of sensation and irritation, will have occasioned the morbid state under consideration. An irritative motion in that case, instead of directly and quietly (with regard to the sentient power) carrying on its vital office, will be disturbed by the morbid influence of sensation, and which will be generating a consciousness of feelings not usually existing, and which may be considered as the offspring of disturbed harmony between the sensitive and irritative functions of life. In this loss of healthful balance, sensation may be experienced wholly unintelligible to ordinary views of disease, making up the infinite variety occasionally occurring in what is denominated nervous affection.

Disorder in the sentient power cannot be confined within given limits: it must, from the nature of the disease, have a range as extensive as the system at large. The influence is not solely transferable by either contiguity or continuity, but but is rapidly diffused in every direction, exerting, in some parts, stronger sensations than in others, according to local and habitual circumstances of susceptibility for diseased impressions. The more usual kind of distress in nervous malady, is that which may be naturally supposed to be excited by irregular actions going on either directly in the nervous structure, or by sympathy in the suffering of other parts. Nature seems to have designed that no material ailment should exist without announcing itself in painful feelings, as a seasonable monition to apply such aid to the occurring disorder as may be necessary. In this view of pain or sentient disorder, it deserves to be regarded rather as a benefit than as a grievance; but, when the disordered state of sentient power goes the afflicting length of rendering such muscular action as is concerned in performing the different visceral functions painful, the case becomes highly aggravated, and is one in which alarm and dismay overwhelm the unhappy sufferer.

The various actions that are incessantly exerting in the different functions of animal life, are by nature involuntary, and do not, in an healthful state, excite in the mind any consciousness of the exertion; but in an highly disordered state of the nervous system, susceptibility by impression may be so keen as to impart a sensation of what is actually going on in the different viscera. It has often occurred to me to observe instances of persons laboring under grievous feelings, which would be referred to the regions at the different vital organs, where no evidence of either visceral or general disease was manifested; where, indeed, the arterial action was regular, appetite sufficient, digestion undisturbed, sleep and other circumstances of health not considerably deranged; and yet the complainings were too correct in the statement given, and too importunate for relief, to admit of the least suspicion as to their reality. That something of imagination may be mingled with complaints of this nature, would be only to admit that it was consistent with their nervous influence to induce more or less of disorder in that faculty.

The general testimony afforded of the fact of disordered sensation going the length of becoming referable to the various visceral actions, received much additional support from a case that has recently fallen under my own observation. In most instances, the patient would describe with an anxiety and accuracy, equally novel and instructive, certain motions obtaining in the brain, lungs, heart, liver, stomach, intestines, in the uterine region, and indeed successively in almost every part of the frame; and in all these descriptions, the utmost persuasion was uniformly held by the patient that the sensations experienced directly emanated from the vital action of the parts in which they were felt. These actions were generally termed by the patient "*wavings* and *heavings*." When the effect was more particularly located, a sense of extreme stricture, inducing deadly oppression, was experienced; when the sensation was more equally diffused, a transient feeling of increased energy of action, and of general lightness of frame, ensue. Amidst this general and local sense of the various actions of life, the utmost severity of disease was experienced and endured for several months. Amusing the attention by a variety of objects, the jerking motion of a carriage, and assiduous friction on different parts of the cutaneous surface, would moderate and even suspend for a time feelings that under circumstances of unmitigated violence were insufferably afflicting. Durable sleep but rarely or ever occurred; that which happened was short and disturbed, and resulted more from exhaustion that from the condition of vital tranquillity naturally disposing to that state. Opium disordered instead of calming, and no description of medicine in any degree availed in overcoming the afflicting agitations that occurred.

During several months this disordered sensation proceeded incessantly, inducing dreadful apprehensions on the part of the patient, either of sudden death, or of eventual loss of intellect. In the various struggles that arose, syncope occasionally happened, in which there appeared to be imminent danger of an extinction of life. At length, what were held to be both the general and local sensations of vital action subsided, when the intellectual function of the brain more particularly suffered. The rational faculty, until then undisturbed, was now overwhelmed by visionary and unfounded views of things. Much correct reasoning was exercised on mistaken notions, which led to erroneous conclusions, and to irrational conduct. This perturbed state of mind gradually subsided, and was followed by an almost immediate and perfect renewal of the intellectual faculty, and a speedy restoration of natural health. It was strikingly remarkable in the case under consideration, that the equal diffusion of morbid sensations over the system, prevented that accumulation of hurtful influence on the brain that could not fail to disturb the rational faculty.

Patients laboring under nervous diseases recount their complaints with endless variety and minuteness; scarcely a disease exists that does not seem to make a part of their ailments. But the reverse of this is the case in maniacal affection; instead of that unhappy state being distinguished by by complainings, the most heroic indifference is shewn to every existing disease. Some predominant thought engrosses the whole attention, rendering the mind inaccessible to all other considerations. Irregular trains of thinking mislead the reason and conduct of the suffering individual, and, according to the objects contemplated, a calm or perturbed frame of mind will be induced; yet but rarely or never, under these circumstances, do morbid sensations pervading the system excite feelings of pain. In these cases a degree of nervous energy obtains in the brain, which probably diffuses a tranquillising influence throughout the system, tending rather to prevent than occasion painful sensations; hence it is usually found, that maniacal disease does not endanger life; that it is rarely connected with bodily ailment; and that its mortal termination, when it has that issue, is affected rather by some violent accident occurring to the function of the brain, in the way of either serous effusion or vascular lesion, suddenly extinguishing life, than from any general affection of the system.

That mania should prove a cure for the morbid sensation here discussed, may be easily conceived; and it is that circumstance that forms the ground of serious dread for the termination of such cases, not only in the apprehension of the patient, but also in that of the medical observer. It would be desirable to avert, if possible, the melancholy result of morbid sensation over the system. The means usually employed for that purpose, are intended to restore the lost energy of the nervous system. The conjoint stimulant influence of medicinal tonics, and mental gratification, is resorted to, as best adapted to raise a fallen and enfeebled state of nervous power throughout the whole frame, to its natural and healthful standard. The best endeavors, however, in this way, generally prove fruitless. The strong nervous excitement induced by the maniacal state, is often more availing; it overcomes the atony and torpor of the general frame, which are the sources of the harassing sensations that at first miserably afflict, and at last madden, the unhappy sufferers. Grief, unappeasable grief, however induced, is the common cause of that enervated and tremulous state of the muscular actions of life, that at length give birth to sensations that are the offspring of disease, and to which the natural state is not at all liable. The dreary scene that unfolds itself in a prospective view of nervous diseases, should afford a timely warning to combat those affections at the outset, not to allow them to become, as it were, inmated with every fibre of the frame, to make every effort to exercise the drooping remains of nervous power, before it shall generate generate feelings that could only be occasioned by a loss of healthful connection between the several faculties of sensibility and of irritability.

If nervous disease really has mania in its train, a truth not to be questioned, it surely has, in all its forms and bearings, the strongest claims on humanity and science to repress its progress and obviate its aggravated mischief. Much might be done at the commencement of sentient disease, but, like all other affections, it derives strength from continuance, until at length it adds the influence of habit to its own peculiar difficulty in being subdued. Were the incipient stages of disease more the objects of medical care, the catalogue of maladies would be neither so heavy nor so incurable as is unhappily now the case.

At the onset of diseases, the morbid character is comparatively simple, and may be correctly understood; but, when aggravated and complicated by protracted duration, the features become obscured, and are rather objects for the exercise of conjecture, than for rational and satisfactory explanation. The strict province of medicine is to prevent the destructive growth of disease, not to waste its resources in attempting to cure incurable ailments. Much may indeed be done by a seasonable application of the therapeutic art; but, if it be delayed too long, nothing but unavailing fear and useless regret await the mortal progress and termination of neglected disease. I am, &c.

*Taunton, Oct.* 7, 1812. ROBERT KINGLAKE.

